# Clinical Evaluation of the Spermatogenic Activity of the Root Extract of Ashwagandha (*Withania somnifera*) in Oligospermic Males: A Pilot Study

**DOI:** 10.1155/2013/571420

**Published:** 2013-11-28

**Authors:** Vijay R. Ambiye, Deepak Langade, Swati Dongre, Pradnya Aptikar, Madhura Kulkarni, Atul Dongre

**Affiliations:** ^1^Mahalaxmi Clinic, Nanaddham, Sulochana Shetty Marg, Sion (E), Mumbai, Maharashtra 400022, India; ^2^Department of Pharmacology, B.V.D.U. Dental College & Hospital, Sector-7, C.B.D., Belpada, Navi Mumbai, Maharashtra 400614, India; ^3^Trupti Hospital & Santati Fertility Center, Thane, Maharashtra 400607, India; ^4^Arya Clinic, Thane, Maharashtra 400601, India; ^5^Arogyadham, Manpada, Thane, Maharashtra 400607, India

## Abstract

Ashwagandha (*Withania somnifera*) has been described in traditional Indian Ayurvedic medicine as an aphrodisiac that can be used to treat male sexual dysfunction and infertility. This pilot study was conducted to evaluate the spermatogenic activity of Ashwagandha root extract in oligospermic patients. Forty-six male patients with oligospermia (sperm count < 20 million/mL semen) were enrolled and randomized either to treatment (*n* = 21) with a full-spectrum root extract of Ashwagandha (675 mg/d in three doses for 90 days) or to placebo (*n* = 25) in the same protocol. Semen parameters and serum hormone levels were estimated at the end of 90-day treatment. There was a 167% increase in sperm count (9.59 ± 4.37 × 10^6^/mL to 25.61 ± 8.6 × 10^6^/mL; *P* < 0.0001), 53% increase in semen volume (1.74 ± 0.58 mL to 2.76 ± 0.60 mL; *P* < 0.0001), and 57% increase in sperm motility (18.62 ± 6.11% to 29.19 ± 6.31%; *P* < 0.0001) on day 90 from baseline. The improvement in these parameters was minimal in the placebo-treated group. Furthermore, a significantly greater improvement and regulation were observed in serum hormone levels with the Ashwagandha treatment as compared to the placebo. The present study adds to the evidence on the therapeutic value of Ashwagandha (*Withania somnifera*), as attributed in Ayurveda for the treatment of oligospermia leading to infertility.

## 1. Introduction

Male infertility accounts for about 50% of human infertility. In 40% to 50% of infertile males, the etiology is unknown [1–7]. The pathophysiology of male infertility could be explained by a number of cellular abnormalities manifesting at the molecular and biochemical levels that result in decreased quality and quantity of sperm in the semen [3–5] and an imbalance in the reproductive hormones. Moreover, it has been widely observed that oligospermia is the single most prevalent cause of reduced male fertility [2, 4].

*Ayurveda*, the traditional system of medicine practiced in India, can be traced back to 6000 Bc [8–11]. For most of this history, Ashwagandha (*Withania somnifera*), also known as “Indian ginseng” due to its rejuvenating effects, has been described in folk medicine as an aphrodisiac and geriatric tonic [12]. It is classified as an “adaptogen,” meaning that this herb assists in combating stress and disease, improving physical strength and metabolism without adverse effects [13–16]. Ashwagandha has been used as a “*rasayana*” in Ayurvedic medicine. In particular, the root of Ashwagandha is regarded as a tonic and aphrodisiac. *Ashwagandha* in the Sanskrit language means “horse's smell” (*ashwa*-horse, *gandha*-smell), probably originating from the odor of its root. The species name *somnifera* means “sleep-inducing” in Latin [17].

Ashwagandha is rich in a wide variety of chemical compounds, such as alkaloids, ergostane steroids, amino acids, and neurotransmitters, which explains its numerous medicinal properties that can directly or indirectly prevent and treat a number of diseases [18–20].

It has been widely documented that, in addition to conventional therapies, many individuals with sexual dysfunction often seek alternative therapies. It is noteworthy that, from ancient times, Ashwagandha  has been used by Ayurvedic practitioners as an aphrodisiac to improve on matters related to infertility and sexual activities. Numerous human and animal studies have validated the aphrodisiac and testosterone-enhancing effects of Ashwagandha [8–11, 21–23].

Different investigators have reported that Ashwagandha is beneficial in the treatment of male infertility [5, 21–23]. Experimental studies have shown that treatment with Ashwagandha induced testicular development and spermatogenesis in immature Wistar rats by directly affecting the seminiferous tubules [5, 24, 25], improved prosexual behaviour of sexually sluggish mice, and increased testicular daily sperm production and serum testosterone level [5].

It has been well documented that high levels of reactive oxygen species (ROS) in the semen induce oxidative damage to the sperm and are associated with abnormal sperm parameters leading to infertility [21, 26–30]. Ashwagandha has been found to counteract the formation of ROS in infertile men [21, 22, 27].

Despite numerous studies that report the efficacy of Ashwagandha in the treatment of various diseases, specific double-blind, randomized, placebo-controlled studies assessing the effectiveness of Ashwagandha in treating male infertility are few and, mostly, lacking critical data on safety and tolerability of the therapy.

Hence, the present study aims to investigate the usefulness of a highly concentrated, full-spectrum root extract of Ashwagandha as a suitable herbal supplement in treating male infertility.

## 2. Subjects and Methods

This two-arm, double-blind, randomized, placebo-controlled, parallel-group study with 1 : 1 random allocation was conducted at five infertility centers in India. The study was conducted in accordance with the good clinical practice guidelines of the Indian Council for Medical Research (ICMR-GCP) and the Declaration of Helsinki and was approved by the “League Health-Independent Ethics Committee.”

### 2.1. Subjects

Sixty-eight infertile males were assessed with regard to eligibility for inclusion in the present study. Forty-six male patients between 22 and 40 years of age with semen factor infertility were enrolled after obtaining informed written consent. All men had a sperm count of 5–20 million/mL, total motility of 10%–30%, with forward motility < 15%, and atypical morphological forms < 70%. All men had a history of regular sexual intercourse over a one-year period with a gynecologically normal female partner with no apparent female infertility.

Men with a total sperm count of <0.5 million/mL or over 20 million/mL were not included. Also, men with primary erectile dysfunction, congenital anomalies, uncontrolled diabetes mellitus, severe hepatic or renal insufficiency, cardiovascular diseases, cerebrovascular accidents, uncontrolled hypertension, or with previous history of cryptorchidism, varicocele and testicular hypertrophy, were excluded from the study. Those with a history of pelvic fractures or prostatectomy or reconstructive or prosthetic surgery on the penis or having total or partly obstructive oligospermia were also excluded. Enrolled patients had not been administered any PDE-V inhibitors (sildenafil, tadalafil, or vardenafil) and glucocorticosteroids within the four weeks prior to enrollment and during the entire course of the study. Patients with known hypersensitivity to Ashwagandha extract were also excluded.

### 2.2. Randomization and Treatments

The study subjects were randomized to either: (i) the placebo-treated group (*n* = 25) or (ii) the Ashwagandha-treated group (*n* = 21). The study subjects in the Ashwagandha-treated group were administered one capsule (containing 225 mg of a high-concentration full-spectrum root extract of the Ashwagandha plant) orally, thrice daily for a period of 12 weeks, whereas, in the placebo-treated group, capsules containing 225 mg of matching placebo were administered similarly.

The Ashwagandha root extract employed in the present study, KSM-66 Ashwagandha (from Ixoreal Biomed Private Ltd., Hyderabad, India), has been extracted with a unique processing technology producing a broad-spectrum phyto-pharmaceutical that potentiates the action of Ashwagandha manifold, providing pan-therapeutic effects. It is noteworthy that although various Ashwagandha powders and extracts are available commercially, there are serious shortcomings in standardization and optimization of Ashwagandha extracts. KSM-66 Ashwagandha is standardized to withanolide content of at least 5% as measured by HPLC. It contains the desired quantum of withanolides and alkaloids, short- and long-chain amino acids (threonine, valine, methionine, isoleucine, lysine, aspartic acid, and arginine), complex sugars including oligosaccharides/fructooligosaccharides, vitamin A, calcium, and iron.

### 2.3. Trial Visits and Assessments

After the screening visit, during the treatment period of 90 days, the subjects were required to present themselves at the trial centers at specified intervals: Visit 1 on Day 30; Visit 2 on Day 60; and Visit 3 on Day 90. The final safety and efficacy assessments were done on Day 90 of the study. Semen analysis and complete physical examination were conducted at baseline and then after 30 days and again after 90 days. Standard manual semen analysis was performed according to WHO guidelines [31, 32]. Hormonal estimations were done for serum testosterone and luteinizing hormone (LH) levels on Day 0 (baseline) and after Day 90 using the chemiluminescence method.

The primary efficacy outcome was the improvement in the semen parameters and serum hormone levels from baseline (Day 0) after 90 days of therapy.

The secondary efficacy outcome was the safety and efficacy of the therapy under investigation. Safety was assessed based on the adverse events recorded during the study. At the end of the study, the four-point Global Assessment Scale for Efficacy (*excellent*,* good*,* satisfactory*, and *poor*) was used for efficacy. The Global Assessment Scale for Tolerability (GATE) was used to assess tolerability to therapy. Compliance was assessed using the tablet count and those who consumed over 80% of tablets were classified as compliant.

### 2.4. Statistical Methods

In this study being of an exploratory nature, the sample size was not based on any distributional assumptions and power calculations.

Efficacy analysis population included all men who completed the study as per the protocol. Safety analysis was done on the intent-to-treat population. The measurement data were expressed as means with one standard deviation. The two groups were compared for change in the sperm count from the baseline using one-way ANOVA with treatment as a factor. The global assessment scale values for efficacy and tolerability to therapy were compared between the two groups by the Mann-Whitney “*U*” test. The obtained results were interpreted as insignificant if the *P* value exceeded 0.05.

## 3. Results

Sixty-eight infertile males were assessed with regard to eligibility for inclusion in the present study. Forty-six were selected for inclusion. This study presents the data of these 46 oligospermic males randomized in a double-blind protocol to either the placebo-treated group (*n* = 25) or the high-concentration, full-spectrum Ashwagandha root extract-treated group (*n* = 21).

The two groups were similar with respect to demographic parameters (Table 1) and all baseline data including semen parameters and serum sexual hormone levels (Table 2).

### 3.1. Semen Parameters

Treatment with the Ashwagandha root extract resulted in a highly significant (*P* < 0.0001) increase in sperm concentration after 90 days of therapy, as compared to the baseline value on Day 0 of the study period (Table 2). The increase was from 9.59 ±  4.37  × 10^6^/mL to 25.61 ± 8.6 × 10^6^/mL, corresponding to a percentage increase of 167%. A statistically significant increase was observed in the semen volume (from 1.74 ± 0.58 mL to 2.76 ± 0.60 mL; *P* < 0.0001) and sperm motility (from 18.62 ± 6.11% to 29.19 ± 6.31%; *P* < 0.0001) on Day 90 as compared to the baseline value on Day 0. These corresponded to increases of 53% and 57%, respectively.

### 3.2. Serum Hormone Levels

**Figure 1:**
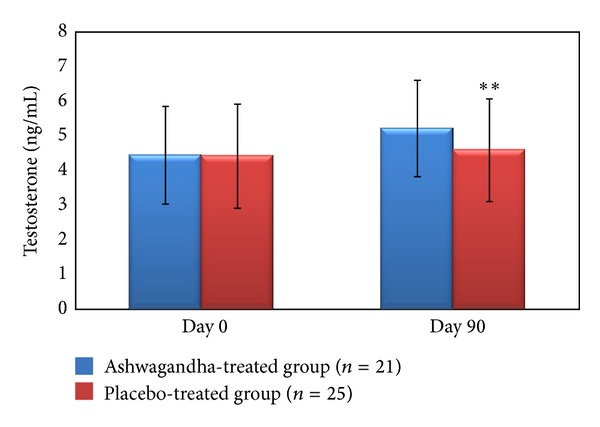
Serum testosterone levels (ng/mL) in the full-spectrum Ashwagandha root extract-treated and placebo-treated study groups including oligospermic males. **P < 0.0001 as compared to baseline values on Day 0 of the study duration of 12 weeks. Values are expressed as mean ± SD.

**Figure 2:**
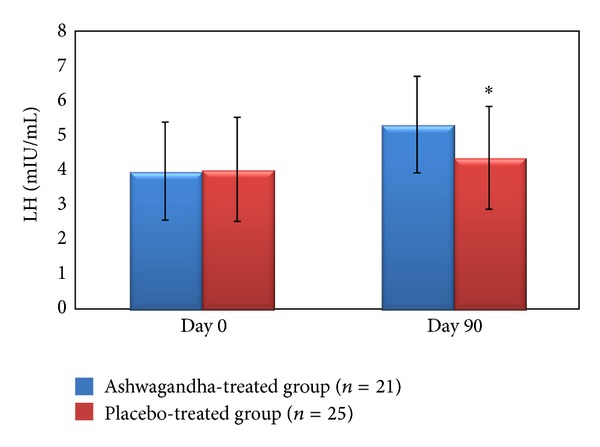
Serum LH (mL IU/mL) in the full-spectrum Ashwagandha root extract-treated and placebo-treated study groups including oligospermic males. *P < 0.001 as compared to baseline values on Day 0 of the study duration (12 weeks). Values are expressed as mean ± SD.

Furthermore, a significantly greater improvement and regulation were observed in serum hormone levels with the Ashwagandha root extract treatment as compared to the placebo treatment. Serum testosterone increased significantly by 17% (from 4.45 ± 1.41 ng/mL to 5.22 ± 1.39 ng/mL; *P* < 0.01) and LH by 34% (from 3.97 ± 1.21 mIU/mL to 5.31 ± 1.33 mIU/mL; *P* < 0.02), following treatment with Ashwagandha root extract, as compared to the baseline (Day 0) values of these parameters (Table 2, Figures 1 and 2).

**Figure 3:**
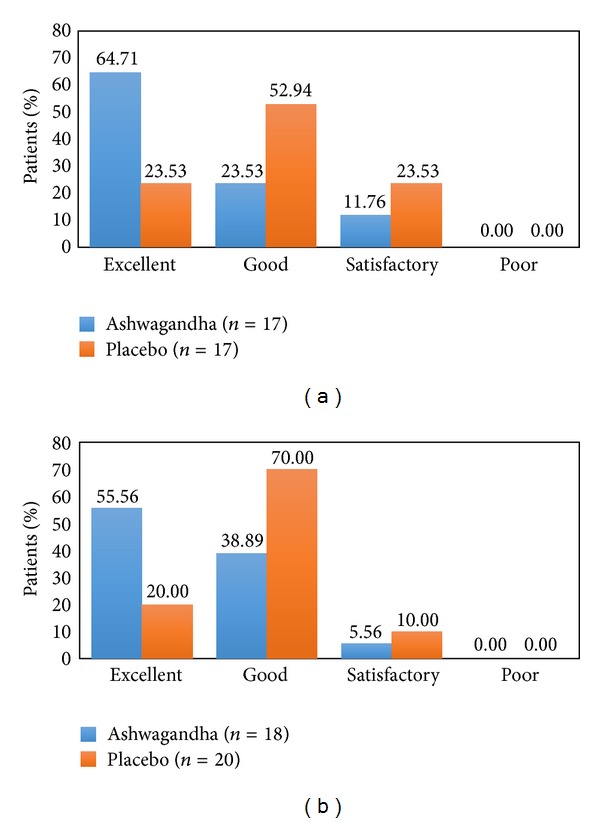
(a) Global Assessment Scale for Tolerability (GATE) by patients. (b) Global Assessment Scale for Tolerability (GATE) by physicians.

Upon evaluation on the Global Assessment Scale for Efficacy (GASE) and Global Assessment Scale for Tolerability (GATE), more patients (68.75%) reported the therapy with Ashwagandha as “excellent” when compared to the placebo (11.76%; Figures 3(a) and 3(b)).

## 4. Discussion

Male infertility accounts for about 50% of human infertility and in 40% to 50% of infertile males the etiology is unknown [1–7]. Numerous studies have demonstrated that compromised semen quality and sperm output are amongst the important causative factors of male infertility [3–5]. Moreover, it has been widely observed that oligospermia is the single most prevalent cause of reduced male fertility [2, 4].

Infertility is defined as the failure to conceive after 12 months of unprotected intercourse with the same partner. Twelve months are the lower reference limit for time to pregnancy by the World Health Organization [6, 31].

Ashwagandha (*Withania somnifera*) is an important medicinal plant that has been used in Ayurvedic medicine for over 6,000 years. In view of its varied and effective therapeutic potential, Ashwagandha has been the subject of considerable modern scientific investigation [8–12]. Ashwagandha has been used for centuries as a “*rasayana*” in Ayurvedic medicine. The root of Ashwagandha is specially regarded as a tonic and aphrodisiac [12–16]. Ashwagandha is often called “Indian ginseng” due to its rejuvenating effects [12, 13]. Nonetheless, the specific effects are not similar to ginseng. Rather than providing restless energy as does ginseng, Ashwagandha often causes relaxation.

In Ayurveda, certain herbal formulas are considered to be rejuvenating [9, 13–15, 18]. These formulas are called “*rasayana*” tonics, taken as a remedy for general weakness and exhaustion, as well as for their stress-relieving qualities.

Chris Kilham, a renowned author, educator and the founder of Medicine Hunter Inc., in accordance with the Indian Materia Medica, emphasized the use of Ashwagandha for general debility, impotence, brain fatigue, low sperm count, nervous exhaustion, and in situations in which general vigor must be restored, as Ashwagandha builds strength from within.

The present study employed a high-concentration, full-spectrum root extract of Ashwagandha, which retains and potentiates the synergism in the whole root.

Extensive clinical and experimental research has been carried out to address possible therapeutic modalities for the treatment of oligospermia utilizing various natural sources of plant and mineral origin as mentioned in Ayurveda and other classical traditional medical texts throughout the world [21–25].

In the present study, treatment with a high-concentration, full-spectrum root extract of Ashwagandha resulted in significantly improved semen parameters in concert with improved and regulated sexual hormone levels in oligospermic males. The analyses of our data indicated significantly increased sperm concentration and overall motility, which are regarded as the most important criteria for normal fertilizing ability of the spermatozoa. Our study outcome showed significant enhancement of the semen volume in the Ashwagandha-treated infertile males.

Our data are in agreement with many investigations reporting improved sperm parameters including sperm concentrations and sperm motility [21–23]. The study by Mahdi et al. [22] compared the effects of Withania in smokers and those with psychological stress, whereas the study by Ahmad et al. [21] focused on the oxidative biomarkers. The observations reported in these studies and the current findings reinforce the beneficial effects of Ashwagandha in maintaining good sperm health and treating “male factor infertility.”

In the present study, treatment with the Ashwagandha root extract resulted in a higher level of testosterone and a concomitant increase in serum levels of LH among infertile men having suboptimal testosterone levels before therapy. Apart from spermatogenesis, testosterone also controls the functional competence of the accessory sex organs, as adequate seminal fluid is necessary for the survival and motility of spermatozoa. Thus, it is postulated that the probable reasons of the increased sperm concentration and motility in the present findings lie in the higher levels of testosterone. These observations have been reported by other workers investigating the fertility-enhancement potential of Ashwagandha and other herbs and minerals [21–23].

Complementing our study, a recent study [22] conducted to assess the effect of Ashwagandha root on semen variables, oxidative biomarkers, and hormone levels among infertile young men aged 25–40 years in India demonstrated increased testosterone and LH among infertile men having suboptimal testosterone levels, compared with the control.

A decrease in testosterone and sperm counts indicates qualitative impairment of spermatogenesis and perhaps defects in sertoli and Leydig cell function [33], pointing toward severe infertility causing reproductive impairment. Thus, testosterone is imperative in aiding the production of sperm. Plant testosterone is much safer than taking an artificial form of testosterone, of which many pharmaceutical products contain [34].

There are reports that gonadal and sexual dysfunction are associated with elevated circulating cortisol levels. Cortisol levels in circulation rise sharply in response to stress followed by a significant drop in testosterone secretion [25]. There are reports that elevated psychological stress is associated with increased oxidative stress that may enhance the generation of reactive oxygen species (ROS).

The hypothalamic-pituitary-gonadal (HPG) axis is known to be involved in stress response and controls spermatogenesis. Hence, disruption of the HPG axis on account of stress results in the failure of the testes to produce adequate levels of testosterone and a normal number of sperms. It is diagnosed by a low sperm count or low serum testosterone levels and reduction in fertility and libido.

Ashwagandha is an effective herbal remedy for stress and infertility. It improves blood circulation throughout the body and enhances sperm quality naturally. Apart from curing sperm problems, intake of Ashwagandha helps in improving the overall health and wellbeing of a person. It relaxes the nerve cells and reduces the occurrence of various health disorders.

The most consistent positive finding of the present study was that decreased fertility in males was ameliorated by Ashwagandha root extract as evidenced by an increase in sperm concentration, ejaculate volume, and motile sperm count and an increase in the serum levels of testosterone.

The use of traditional or complementary/alternative medicine (CAM) for health care has been increasingly described in medical and science reports [35]. There has been an effort in recent years to evaluate the pharmacological properties of Ashwagandha, which has resulted in a better understanding of its therapeutic potential.

Nonetheless, if medical professionals are to prescribe herbal remedies for male infertility or any other medical condition, previous rigorous scientific investigations documenting their safety and efficacy from a Western scientific perspective are required. The outcomes of the present study provide evidence for the safety, efficacy, and tolerability of therapy with Ashwagandha root extract.

The present study suggests potential role of high-concentration, full-spectrum root extract of Ashwagandha in treating male infertility, which needs further exploration.

## Figures and Tables

**Table 1:** Demography and baseline data of the study subjects.

	Placebo (*n* = 25) mean ± SD	Ashwagandha (*n* = 21) mean ± SD
Age (yr)	35.28 ± 5.49	32.38 ± 4.31
Height (cm)	167.13 ± 7.53	165.89 ± 8.55
Weight (kg)	74.32 ± 14.52	70.05 ± 11.22
Pulse (per min)	80.04 ± 8.06	79.78 ± 6.96
Respiratory rate (per min)	16.92 ± 2.45	17.39 ± 1.91
Systolic blood pressure (mm Hg)	123.84 ± 9.50	128.00 ± 8.03
Diastolic blood pressure (mm Hg)	80.16 ± 4.28	80.33 ± 3.65
Body temperature (°F)	98.04 ± 0.36	97.83 ± 0.38

**Table 2:** Semen profile of the Ashwagandha root extract-treated and placebo-treated oligospermic males.

	Placebo-treated group (*n* = 25)			Ashwagandha-treated group (*n* = 21)		
	Day 0	Day 60	Day 90	Day 0	Day 60	Day 90
Sperm concentration (×10^6^/mL)	10.24 ± 2.82	12.42 ± 4.75	13.23 ± 7.74	9.59 ± 4.37	18.8 ± 5.7^#^	25.61 ± 8.6^#^
Semen volume (mL)	1.88 ± 0.65	2.3 ± 0.58	2.25 ± 0.41	1.74 ± 0.58	2.56 ± 0.7*	2.76 ± 0.6^#^
Sperm motility (%)	18.6 ± 5.41	18.87 ± 5.86	20.27 ± 5.97	18.62 ± 6.11	26.04 ± 5.6^#^	29.19 ± 6.31^#^
Serum testosterone ng/mL	4.42 ± 1.50	—	4.59 ± 1.48	4.45 ± 1.41	—	5.22 ± 1.39
Serum LH mIU/mL	4.02 ± 1.20	—	4.35 ± 1.28	3.97 ± 1.21	—	5.31 ± 1.33

*P < 0.05, ^#^ *P* < 0.0001 as compared to the baseline values on Day 0 of the study duration (12 weeks). Values are expressed as mean ± SD.
